# Insulin Resistance is Associated with Cognitive Decline Among Older Koreans with Normal Baseline Cognitive Function: A Prospective Community-Based Cohort Study

**DOI:** 10.1038/s41598-017-18998-0

**Published:** 2018-01-12

**Authors:** Sung Hye Kong, Young Joo Park, Jun-Young Lee, Nam H. Cho, Min Kyong Moon

**Affiliations:** 1 Department of Internal Medicine, Seoul National University College of Medicine, Seoul, Republic of Korea; 2 Department of Psychiatry and Behavioral Science, Seoul National University Boramae Medical Center, Seoul, Republic of Korea; 3 Department of Preventive Medicine, Ajou University School of Medicine, Suwon, Republic of Korea; 4 Department of Internal Medicine, Seoul National University Boramae Medical Center, Seoul, Republic of Korea

## Abstract

We evaluated whether metabolic factors were associated with cognitive decline, compared to baseline cognitive function, among geriatric population. The present study evaluated data from an ongoing prospective community-based Korean cohort study. Among 1,387 participants who were >65 years old, 422 participants were evaluated using the Korean mini-mental status examination (K-MMSE) at the baseline and follow-up examinations. The mean age at the baseline was 69.3 ± 2.9 years, and 222 participants (52.6%) were men. The mean duration of education was 7.1 ± 3.6 years. During a mean follow-up of 5.9 ± 0.1 years, the K-MMSE score significantly decreased (−1.1 ± 2.7 scores), although no significant change was observed in the homeostasis model assessment of insulin resistance (HOMA-IR) value. Participants with more decreased percent changes in K-MMSE scores had a shorter duration of education (*p* = 0.001), older age (*p* = 0.022), higher baseline K-MMSE score (*p* < 0.001), and increased insulin resistance (∆HOMA-IR, *p* = 0.002). The correlation between the percent changes in K-MMSE and ∆HOMA-IR values remained significant after multivariable adjustment (*B* = −0.201, *p* = 0.002). During a 6-year follow-up of older Koreans with normal baseline cognitive function, increased insulin resistance was significantly correlated with decreased cognitive function.

## Introduction

The increasing geriatric population is associated with increasing prevalence of dementia and cognitive dysfunction in many countries, including Korea^1–3^. The World Health Organization has reported that 47.5 million people had dementia in 2016, with global totals projected to reach 75.6 million people in 2030 and 135.5 million people in 2050^4^. Dementia in geriatric populations affects the individuals’ and their families’ quality of life, which creates a societal burden, and the global estimated economic burden was approximately 604 billion US dollars in 2010^4^. Therefore, it would be helpful to identify and address modifiable factors that can help to reduce the global burden of dementia.

Epidemiological studies have revealed associations of cognitive impairment and/or dementia with type 2 diabetes mellitus^5,6^. Insulin resistance, hyperglycemia, and obesity are probable mechanistic links in this association. Few longitudinal studies have evaluated this issue, although many cross-sectional studies have confirmed the relationship between insulin resistance and cognitive decline^7–10^. One longitudinal study evaluated middle-aged adults (45–64 years old at baseline in the Atherosclerosis Risk in Communities cohort), and revealed that baseline hyperinsulinemia was associated with a rapid decline in cognitive function^7^. Another longitudinal nationwide population-based survey revealed that higher baseline HOMA-IR and fasting insulin levels were associated with a greater decline in verbal fluency^8^. However, to the best of our knowledge, no studies have evaluated the effects of longitudinal changes in insulin resistance on cognitive function.

Although clinical studies of patients with diabetes have demonstrated that hyperglycemia is associated with cognitive dysfunction, it remains controversial whether hyperglycemia is associated with cognitive dysfunction among older individuals or among individuals with normal glucose tolerance or prediabetes^9–12^. Among patients with impaired glucose tolerance, there is very little evidence regarding a relationship between impaired glucose tolerance and cognitive impairment^12,13^. However, among participants with normal glucose tolerance, poor glucose tolerance was associated with cognitive impairment^12,13^. The Leiden 85-plus Study prospectively evaluated 599 individuals from the age of 85 years and revealed that HbA1c concentrations were not associated with cognitive dysfunction^9^.

It has also been suggested that obesity is a risk factor for cognitive dysfunction, although this relationship remains unclear, as the few longitudinal studies have provided conflicting results^14–16^.

As geriatric populations are consistently growing, it is important to determine whether metabolic factors are related to cognitive dysfunction among older individuals, and whether addressing these metabolic factors can help prevent cognitive dysfunction. In the present prospective community-based cohort study, we aimed to identify metabolic factors that were associated with cognitive decline among older individuals with normal baseline cognitive function.

## Methods

### Study population

The present study evaluated data from the Ansung cohort study, which is a prospective community-based study that began in 2001 and is supported by National Genome Research Institute (Korean Centers for Disease Control and Prevention, Cheongju, Korea). That study is a part of the Korean Genome Epidemiology Study, which is a community-based epidemiological survey of Korean individuals who are 40–69 years old. The Ansung study recruited residents of Ansung who had lived in the surveyed region for ≥6 months. According to the 2000 census, Ansung is a rural community with 132,906 residents. Detailed information regarding the Ansung study’s selection criteria and sampling plan has been published previously^17,18^, and the study’s protocol was approved by the institutional review board of the Korean Centers for Disease Control and Prevention and the study was carried out in accordance with the protocol. Informed consent was obtained from all participants and/or their legal guardians. All methods were performed in accordance with the relevant guidelines and regulations.

**Figure 1:**
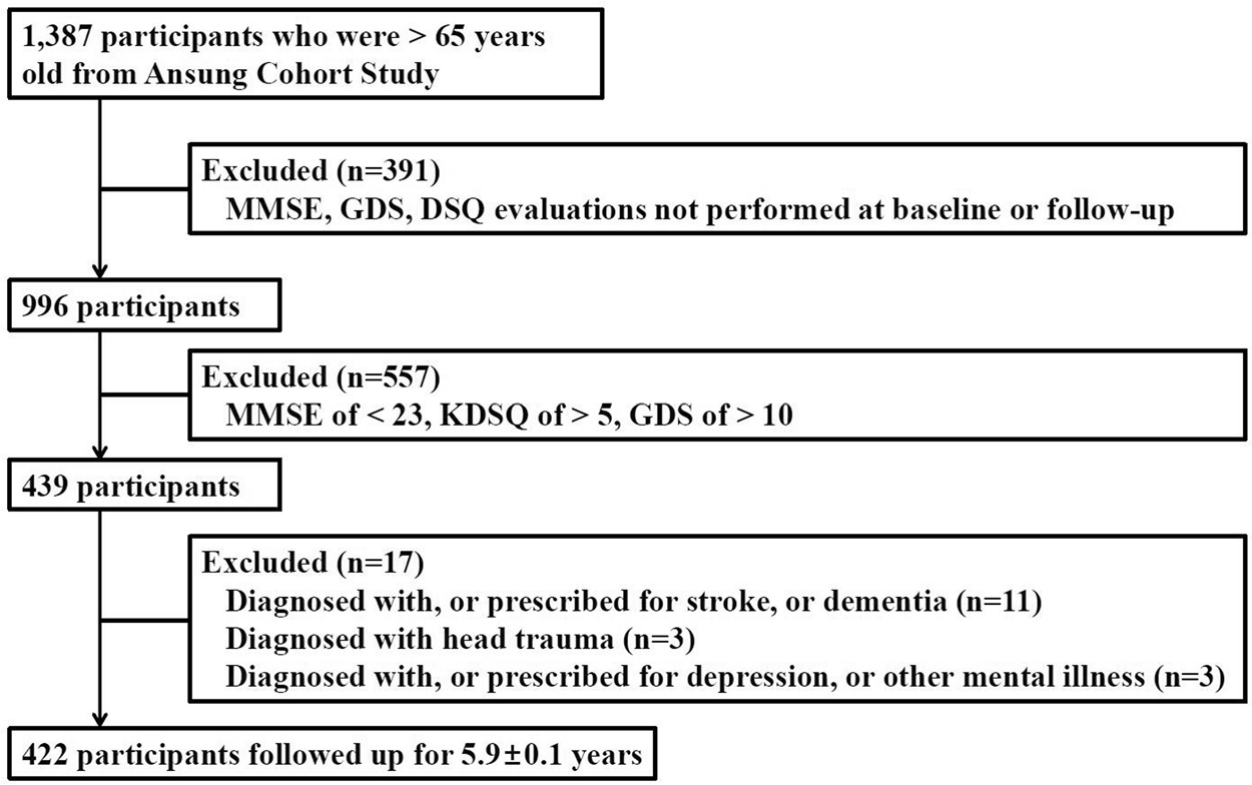
Flow chart showing selection of the study population from Ansung cohort study.

The Ansung cohort study evaluated 1,387 participants who were >65 years old, completed a baseline examination in 2001, and were surveyed biennially until 2014. Data from these participants were considered for inclusion in the present study. However, we excluded 391 participants because they did not complete the baseline or follow-up Korean mini-mental status examination (K-MMSE), the Korean geriatric depression score tool (GDS-K), or the Korean dementia screening questionnaire (KDSQ). In addition, we excluded 557 participants with a K-MMSE score of <23, a KDSQ score of >5, or a GDS-K score of >10. Participants with impaired baseline cognitive function (low K-MMSE score or high KDSQ score) were excluded because we only intended to evaluate individuals with normal baseline cognitive function. Participants with high GDS scores were excluded to minimize the influence of depressiveness on the MMSE results, as depression can be associated with low MMSE scores^19^. Furthermore, we excluded 17 participants who had been diagnosed with stroke, dementia, depression, or head trauma. Thus, data from 422 eligible participants were included in the final analyses (Fig. 1). The mean follow-up duration was 5.9 ± 0.1 years.

### Assessing cognitive impairment and depression

Cognitive impairment and depression were measured at the baseline and follow-up examinations. Cognitive impairment was evaluated using the K-MMSE and KDSQ tools. The 30-item K-MMSE was specifically developed and validated for assessing the general cognitive function of older Korean individuals^20^. The results are scored from 0 to 30 points, with scores of ≥23 points indicating normal cognition, scores of 17–22 points indicating mild cognitive impairment, and scores of <17 points indicating moderate-to-severe impairment. The 15-item KDSQ is a sensitive test for early dementia screening, and the results are not influenced by age or educational level^21^. The results are scored from 0 to 15 points, with scores of >5 points considered suggestive of cognitive impairment. Depressive symptoms were assessed using the 15-item GDS-K^22^. The results are scored from 0 to 15 points, with scores of >10 points considered suggestive of depressive mood. Changes in the K-MMSE, GDS-K, and KDSQ scores were calculated as:

1 $${\rm{\Delta }}K \mbox{-} \mathrm{MMSE}=\mathrm{follow} \mbox{-} \mathrm{up}\,K \mbox{-} \mathrm{MMSE}-{\rm{baseline}}\,K \mbox{-} \mathrm{MMSE}$$

2 $${\rm{Percent}}\,{\rm{changes}}\,{\rm{in}}\,K \mbox{-} \mathrm{MMSE}={\rm{\Delta }}K \mbox{-} \mathrm{MMSE}/{\rm{baseline}}\,K \mbox{-} \mathrm{MMSE}$$

3 $${\rm{\Delta }}\mathrm{GDS} \mbox{-} K=\mathrm{follow} \mbox{-} \mathrm{up}\,\mathrm{GDS} \mbox{-} K-{\rm{baseline}}\,\mathrm{GDS} \mbox{-} K$$

4 $${\rm{\Delta }}\mathrm{KDSQ}=\mathrm{follow} \mbox{-} \mathrm{up}\,{\rm{KDSQ}}-{\rm{baseline}}\,{\rm{KDSQ}}$$

### Measuring anthropometric parameters

Face-to-face or telephone interviews were used to obtain data regarding the participants’ age, sex, duration of education, medical history, alcohol consumption, and smoking status. Former smokers were defined as individuals who had smoked >5 packs of cigarettes during their lifetime, and participants were defined as having quit smoking if they had stopped smoking ≥6 months before the baseline examination. Former drinkers were defined as individuals who had consumed 5 g of ethanol/day, and participants were defined as having quit drinking if they had stopped consuming alcohol ≥6 months before the baseline examination. Medical histories of diabetes, hypertension, stroke, dementia, head trauma, depression, and other mental illness were identified based on self-reported diagnoses.

The participants’ height and body weight were measured using the standard methods (scale and wall-mounted extensometer) while the participants were wearing light-weight clothes. Body mass index (BMI) was calculated as weight divided by height squared (kg/m^2^). Changes in BMI (∆BMI) were calculated as:

5 $${\rm{Equation}}\,4:{\rm{\Delta }}{\rm{BMI}}=\mathrm{follow} \mbox{-} \mathrm{up}\,{\rm{BMI}}-{\rm{baseline}}\,{\rm{BMI}}$$

### Laboratory testing and homeostasis model assessment of insulin resistance calculation

All participants fasted for at least 14 h before undergoing the blood sampling. Plasma specimens were separated using centrifugation (2,000 rpm for 20 min at 4 °C) and tested immediately. Plasma glucose concentrations were measured using the hexokinase method (ADVIA 1650 Auto Analyzer; Bayer, Leverkusen, Germany), and plasma insulin concentrations were measured using the IRMA test kit (bioSource Europe S.A., Niverlles, Belgium). Fasting concentrations of total cholesterol, high-density lipoprotein cholesterol (HDL-C), low-density lipoprotein cholesterol (LDL-C), and triglycerides were measured enzymatically using the Hitachi 747 chemistry analyzer (Hitachi, Tokyo, Japan). Concentrations of HbA1c were evaluated using high-performance liquid chromatography with a Bio-Rad Variant II HbA1c analyzer (Bio-Rad, Montreal, Quebec, Canada). Consenting participants underwent apolipoprotein E genotyping using the methods of Hixson and Vernier^23^, and the results were categorized based on the presence or absence of the ε4 allele. HOMA-IR^24^, ∆HOMA-IR, ∆HbA1c, and ∆fasting insulin were calculated as:

6 $$\mathrm{HOMA} \mbox{-} \mathrm{IR}=({\rm{fasting}}\,{\rm{plasma}}\,{\rm{insulin}}\,[{\rm{\mu }}\mathrm{IU}/{\rm{mL}}]\times {\rm{fasting}}\,{\rm{plasma}}\,{\rm{glucose}}[{\rm{mg}}/{\rm{dL}}]\times 0.0555)/22.5$$

7 $${\rm{\Delta }}\mathrm{HOMA} \mbox{-} \mathrm{IR}=\mathrm{follow} \mbox{-} \mathrm{up}\,\mathrm{HOMA} \mbox{-} \mathrm{IR}-{\rm{baseline}}\,\mathrm{HOMA} \mbox{-} \mathrm{IR}$$

8 $${\rm{\Delta }}\mathrm{HbA}1{\rm{c}}=\mathrm{follow} \mbox{-} \mathrm{up}\,{\rm{HbA}}1{\rm{c}}-{\rm{baseline}}\,{\rm{HbA}}1{\rm{c}}$$

9 $${\rm{\Delta }}\mathrm{fasting}\,{\rm{insulin}}=\mathrm{follow} \mbox{-} \mathrm{up}\,{\rm{fasting}}\,{\rm{insulin}}-{\rm{baseline}}\,{\rm{fasting}}\,{\rm{insulin}}$$

### Statistical analysis

Normally distributed data were presented as mean ± standard deviation, non-normally distributed data were reported as median (interquartile range [IQR]), and categorical data were reported as number (%). The participants’ characteristics at the baseline and 6-year follow-up examinations were compared using the paired *t*-test, Wilcoxon signed-rank test, and Mann-Whitney *U* test, as appropriate. Pearson’s correlation coefficient was used to estimate the relationship between cognitive function and the related parameters. *B* refers to standardized beta value. Associations of cognitive function with the other factors were analyzed using multivariable linear regression models. Model 1 was adjusted for age, sex, baseline K-MMSE score, education duration, and baseline GDS-K score. Model 2 was adjusted for the factors in model 1 plus smoking status, history of diabetes, history of hypertension, and BMI. Model 3 was adjusted for the factors in model 2 plus the apolipoprotein E ε4 genotype status. Differences were considered statistically significant at *p*-values of <0.05, and all analyses were performed using IBM SPSS software (version 22.0; IBM Corp., Armonk, NY, USA).

### Data Availability

The datasets generated during and/or analyzed during the current study are available from the corresponding author on reasonable request.

## Results

### Baseline characteristics

The participants’ characteristics at the baseline and follow-up examinations are shown in Table 1. The mean follow-up duration was 5.9 ± 0.1 years, the mean age at baseline was 69.3 ± 2.9 years, and 222 participants (52.6%) were men. The mean education duration was 7.1 ± 3.6 years. At baseline, 115 participants (27.3%) had hypertension, compared to 207 participants (49.1%) at the follow-up. At baseline, 89 participants (21.1%) had diabetes, compared to 113 participants (26.8%) at the follow-up. The K-MMSE scores decreased significantly from 26.5 ± 1.9 at baseline to 25.4 ± 2.9 at the follow-up (∆K-MMSE, −1.1 ± 2.7, percent changes in K-MMSE, −4.1 ± 10.3%). The KDSQ scores increased significantly (median increase: 1.0, IQR: –1.0 to 4.0). The GDS-K scores also increased significantly at the follow-up. The laboratory test results from the follow-up revealed a significant decrease in the LDL-C concentration and significant increases in the HDL-C and creatinine concentrations. The laboratory results for lipid profile, including LDL-C, HDL-C, and triglycerides, were only analyzed for 371 participants who were not receiving dyslipidemia treatment. No significant differences were observed in the values for HOMA-IR, fasting insulin, and HbA1c. Seventy-two participants (17.1%) were found to have the apolipoprotein E **ε**4 genotype (Table 1).

**Table 1:** Characteristics of the participants at baseline and follow-up.

	**Baseline (n = 422)**	**Follow-up (n = 422)**	***p*** **-value**
Age, years	69.3 ± 2.9	75.3 ± 2.9	<0.001
Male sex, n (%)	222 (52.6)		
Education duration, years	7.1 ± 3.6		
Hypertension, n (%)	115 (27.3)	207 (49.1)	
Diabetes, n (%)	89 (21.1)	113 (26.8)	
Ever smoker, n (%)	151 (35.8)	167 (39.6)	
Current alcohol intake, n (%)	198 (46.9)	194 (46.0)	
BMI, kg/m^2^	23.9 ± 3.1	23.7 ± 3.3	0.015
∆BMI, kg/m^2^	−0.17 ± 1.38		
K-MMSE baseline, score	26.5 ± 1.9	25.4 ± 2.9	<0.001
∆K-MMSE, score	−1.1 ± 2.7		
Percent changes in K-MMSE	−4.1 ± 10.3		
HOMA-IR baseline	1.78 (1.34, 2.56)	1.79 (1.31, 2.56)	0.970
∆HOMA-IR	−0.02 (−0.56, 0.56)		
KDSQ, score	2.0 (1.0, 4.0)	3.0 (1.0, 6.0)	<0.001
∆KDSQ, score	1.0 (−1.0, 4.0)		
GDS-K score	2.0 (1.0, 4.0)	2.0 (0, 5.0)	<0.001
∆GDS-K score	0.6 ± 3.5		
Fasting insulin, μIU/L	8.8 ± 5.4	8.8 ± 4.2	0.845
∆fasting insulin, μIU/L	−0.1 ± 5.9		
HbA1c, % (mmol/mol)	5.8 ± 0.8 (40 ± 3)	5.8 ± 0.7 (40 ± 3)	0.616
∆HbA1c, % (mmol/mol)	0 ± 0.6 (2.2 ± 2.2)		
LDL-C, mg/dL^a^	120.0 ± 28.7	109.5 ± 29.1	<0.001
HDL-C, mg/dL^a^	43.1 ± 10.5	44.3 ± 11.8	0.041
Triglycerides, mg/dL^a^	128.4 ± 62.6	121.8 ± 58.2	0.055
Creatinine, mg/dL	0.9 ± 0.2	1.1 ± 0.3	<0.001
APOE ε4 genotype, %	72 (17.1)		

K-MMSE, Korean mini mental status examination; KDSQ, Korean dementia screening questionnaire; GDS-K, Korean geriatric depression scale; APOE ε4, apolipoprotein ε4; LDL-C, low-density lipoprotein cholesterol; HDL-C, high-density lipoprotein cholesterol.

Continuous variables are reported as mean ± standard deviation for normally distributed variables, while median (interquartile range) is used for HOMA-IR, ∆HOMA-IR, KDSQ, and GDS-K (non-normally distributed variables), and n (%) is used for categorical variables. The ∆ values refer to the change between baseline and follow-up.

^a^Lipid profile was only evaluated among participants who were not receiving dyslipidemia treatment (n = 371).

### Correlations of K-MMSE with factors at baseline

Pearson’s correlation analyses revealed that lower K-MMSE values were correlated with shorter education durations (*r* = 0.393, *p* < 0.001), higher KDSQ scores (*r* = −0.129, *p* = 0.008), and higher GDS-K scores (*r* = −0.128, *p* = 0.008). Baseline K-MMSE values were not significantly correlated with age, baseline BMI, ∆BMI, ∆KDSQ score, ∆GDS-K score, baseline HOMA-IR, ∆HOMA-IR, baseline fasting insulin, ∆fasting insulin, baseline HbA1c, ∆HbA1c, baseline lipid profiles, or creatinine (Table 2).

**Table 2:** Correlations between baseline K-MMSE and related factors.

	**Correlation coefficient (** ***r*** **)**	***p*** **-value**
Age, years	−0.016	0.746
Education, years	0.393	<0.001
Baseline BMI, kg/m^2^	−0.058	0.240
∆BMI, kg/m^2^	0.005	0.927
Baseline KDSQ, score	−0.129	0.008
∆KDSQ, score	0.031	0.523
Baseline GDS-K, score	−0.128	0.008
∆GDS-K, score	0.028	0.569
Baseline HOMA-IR	0.006	0.902
∆HOMA-IR	−0.027	0.586
Baseline fasting insulin, μIU/L	−0.019	0.703
∆fasting insulin, μIU/L	−0.028	0.566
Baseline HbA1c, % (mmol/mol)	0.034	0.481
∆HbA1c, % (mmol/mol)	−0.048	0.326
Baseline LDL-C, mg/dL^a^	−0.002	0.961
Baseline HDL-C, mg/dL^a^	−0.031	0.526
Baseline triglycerides, mg/dL^a^	−0.020	0.974
Baseline creatinine, mg/dL	0.021	0.840

K-MMSE, Korean mini mental status examination; KDSQ, Korean dementia screening questionnaire; GDS-K, Korean geriatric depression scale; LDL-C, low-density lipoprotein cholesterol; HDL-C, high-density lipoprotein cholesterol. The ∆ values refer to the change between baseline and follow-up.

^a^Lipid profile was only evaluated among participants who were not receiving dyslipidemia treatment (n = 371).

### Correlations of percent changes in K-MMSE with related factors

Pearson’s correlation analyses revealed that a greater decrease in K-MMSE between baseline and follow-up was correlated with older age (*r* = –0.102, *p* = 0.037), shorter education duration (*r* = 0.168, *p* = 0.001), higher baseline K-MMSE score (*r* = –0.187, *p* < 0.001), and increases in HOMA-IR values (*r* = –0.155, *p* = 0.001) and fasting insulin levels between baseline and follow-up (*r* = –0.160, *p* = 0.001). The percent changes in K-MMSE values were not significantly correlated with the values for baseline BMI, ∆BMI, baseline KDSQ score, ∆KDSQ score, GDS-K score, ∆GDS-K score, baseline HOMA-IR, baseline fasting insulin, baseline HbA1c, ∆HbA1c, lipid profiles, or creatinine (Table 3).

**Table 3:** Correlations of percent changes in K-MMSE between baseline and follow-up and related factors.

	**Correlation coefficient (** ***r*** **)**	***p*** **-value**
Age, years	−0.102	0.037
Education, years	0.168	0.001
K-MMSE baseline, score	−0.187	<0.001
BMI, kg/m^2^	−0.012	0.810
∆BMI, kg/m^2^	0.044	0.370
KDSQ, score	0.042	0.388
∆KDSQ, score	−0.082	0.093
GDS-K, score	0.006	0.909
∆GDS-K, score	−0.081	0.096
HOMA-IR baseline	0.057	0.247
∆HOMA-IR	−0.155	0.001
Fasting insulin, μIU/L	0.073	0.133
∆fasting insulin, μIU/L	−0.160	0.001
HbA1c, % (mmol/mol)	−0.052	0.289
∆HbA1c, % (mmol/mol)	0.040	0.412
LDL-C, mg/dL^a^	0.044	0.366
HDL-C, mg/dL^a^	−0.020	0.687
Triglyceride, mg/dL^a^	0.037	0.447
Creatinine, mg/dL	0.050	0.308

K-MMSE, Korean mini mental status examination; KDSQ, Korean dementia screening questionnaire; GDS-K, Korean geriatric depression scale; LDL-C, low-density lipoprotein cholesterol; HDL-C, high-density lipoprotein cholesterol. The ∆ values refer to the change between baseline and follow-up.

^a^Lipid profile was only evaluated among participants who were not receiving dyslipidemia treatment (n = 371).

### Multivariable linear regression models for percent changes in K-MMSE and the hyperinsulinemia variables

After adjusting model 1 for age, sex, baseline K-MMSE score, education duration, and baseline GDS-K score, we observed that an increase in HOMA-IR was correlated with a reduction in K-MMSE between baseline and follow-up (*B* = −0.139, *p* = 0.003). After adjusting for the variables in model 2 (model 1 plus smoking status, distort of diabetes, history of hypertension, and BMI), we observed that the negative correlation between change of HOMA-IR and K-MMSE remained significant (standardized beta [*B*] = −0.137, *p* = 0.004). After adjusting for the variables in model 3 (model 2 plus apolipoprotein E ε4 genotype status), the correlation between change of HOMA-IR and K-MMSE remained significant (*B* = −0.138, *p* = 0.004). Percent changes in K-MMSE values were not correlated with ∆BMI, ∆GDS-K scores, and ∆HbA1c before and after adjusting covariates before and after adjusting covariates (Table 4).

**Table 4:** Multivariable linear regression models of percent changes in K-MMSE between baseline and follow-up and their correlations with metabolic factors.

	***B*** **of ∆HOMA-IR**	**95% confidence interval**	***p-value***
Unadjusted	−0.155	−1.444, −0.349	0.001
Model 1	−0.139	−1.326, −0.281	0.003
Model 2	−0.137	−1.304, −0.257	0.004
Model 3	−0.138	−1.307, −0.256	0.004
	***B*** **of ∆BMI**	**95% confidence interval**	***p-value***
Unadjusted	0.044	−0.389, 1.044	0.370
Model 1	0.009	−0.630, 0.758	0.856
Model 2	0.015	−0.597, 0.822	0.755
Model 3	0.018	−0.586, 0.850	0.717
	***B*** **of ∆GDS-K**	**95% confidence interval**	***p-value***
Unadjusted	−0.081	−0.517, 0.043	0.096
Model 1	−0.079	−0.516, 0.051	0.107
Model 2	−0.073	−0.494, 0.074	0.146
Model 3	−0.074	−0.501, 0.073	0.143
	***B*** **of ∆HbA1c**	**95% confidence interval**	***p-value***
Unadjusted	0.040	−0.959, 2.334	0.412
Model 1	0.009	−1.422, 1.729	0.849
Model 2	0.016	−1.324, 1.875	0.735
Model 3	0.016	−1.341, 1.869	0.746

K-MMSE, Korean mini mental status examination; BMI, body mass index; GDS-K, Korean geriatric depression scale.

Multivariable linear regression analysis was done. *B* refers to standardized beta value. Model 1 adjusts for age, sex, baseline K-MMSE, education duration, baseline GDS-K. Model 2 adjusts for model 1 factors and additionally adjusts for smoking status, history of diabetes and hypertension, and BMI. Model 3 adjusts for model 2 factors and additionally adjusts for APOE ε4 genotype status.

## Discussion

This community-based prospective cohort study of older individuals with normal cognitive function revealed that cognitive decline was associated with a 6-year increase in insulin resistance, after adjusting for age, sex, baseline K-MMSE, education duration, baseline GDS-K, smoking status, history of diabetes, history of hypertension, BMI, and apolipoprotein E ε4 genotype status. Among the various metabolic factors, cognitive dysfunction was associated with changes in insulin resistance, but not with existing or changing hyperglycemia or obesity. Furthermore, greater cognitive declines were observed for participants with greater increases in insulin resistance.

Most previous studies investigating the association of insulin resistance with cognitive dysfunction have used a cross-sectional design, and few longitudinal studies have been performed^7–10^. During a 6-year follow-up, baseline hyperinsulinemia among middle-aged adults (45–64 years old at baseline) in the Atherosclerosis Risk in Communities cohort was associated with greater cognitive decline^7^. In addition, a recent 11-year Finnish nation-wide population-based survey revealed that higher baseline HOMA-IR and fasting insulin values independently predicted a greater decline in verbal fluency^8^. Furthermore, our findings are consistent with previous findings that insulin resistance is related to cognitive decline^7,10,25–28^. Moreover, we evaluated the relationship between changes in insulin resistance and cognitive decline, and the results suggest that controlling insulin resistance could help prevent cognitive decline in the older population.

Insulin receptors are widely distributed throughout the brain, which suggests that the brain is a major insulin target^29^. Furthermore, insulin resistance is observed in the neuronal cells of patients with Alzheimer’s disease (AD), which suggests that insulin resistance may cause neuronal cell dysfunction and lead to cognitive dysfunction and dementia^30,31^. Interestingly, mice receiving a high-fat diet exhibited impaired binding to brain insulin receptors^32^, which agrees with the findings from the brains of patients with early-stage AD. Therefore, neurons in the brain might be damaged by insulin receptor dysfunction. Moreover, peripheral insulin resistance could induce neuronal damage though amyloid beta and cytokines, as peripheral hyperinsulinemia can increase amyloid beta concentrations^33^ and plasma and cerebrospinal fluid concentrations of interleukin-6 and tumor necrosis factor alpha^34^. The increased concentrations of amyloid beta and inflammatory cytokines could induce neuronal loss, amyloid beta plaques, and neurofibrillary tangles^35^.

The present study did not detect any significant relationship between the baseline HOMA-IR and K-MMSE values. Although many previous studies have identified a positive association between insulin resistance and cognitive dysfunction^7,25,27,28^, other studies did not detect any significant association^36,37^. These discrepancies may be related to differences in the study populations and cognitive function assessment methods. For example, studies with positive results generally evaluated individuals who did not have baseline cognitive dysfunction^28^, were relatively young^7^ or were women^38,39^. In addition, insulin resistance may mainly affect executive dysfunction, which is best evaluated using the Trail Making Test^39,40^ and/or verbal fluency^38,41^, rather than tools that consider all brain function domains. However, the present study did not detect any significant correlation between cognitive function and insulin resistance when we only analyzed women. Therefore, it appears that methodology differences are more important, rather than sex-based differences.

It is suggested that hyperglycemia is associated with poor cognitive outcomes. It has been shown in both cross-sectional studies^42^ and prospective studies^43^, although there are conflicting results. The present study failed to detect a significant correlation between hyperglycemia and cognitive function or change in cognitive function. This may be because the HbA1c range was relatively narrow (caused by the community-based design), while most studies with positive results evaluated patients with diabetes. Moreover, hyperglycemia mainly affects processing speed, attention, and visual-spatial processing^44^, which may not be accurately assessed using only the MMSE tool.

Although many studies have revealed an association between obesity and the risk of dementia^12,14–16,43,44^, the association appears to be complex. For example, some reports have indicated that higher BMI was associated with less cognitive decline in a cognitively unimpaired community-dwelling population, and there are reports of increased dementia risk for both obese and underweight people^12,15,45^. In addition, studies have demonstrated that midlife obesity is more strongly related to dementia, compared to obesity among older people^12,14^. We did not detect any significant association between BMI and K-MMSE or the change in K-MMSE. This may be related to the participants’ relatively normal BMI (mean 23.9 kg/m^2^), or midlife BMI having a greater effect on the cognitive function of older individuals, compared to current BMI.

The present study evaluated the participants’ serum cholesterol concentrations after excluding individuals who were receiving dyslipidemia medication. However, the follow-up examinations revealed lower concentrations of triglycerides and LDL-C, and higher concentrations of HDL-C. These changes may be partially explained by aging-related changes^46^, and enrollment in the cohort may also have improved the participants’ lifestyle, as it was accompanied by an increase in HDL-C concentrations. Furthermore, there is the possibility of recall bias regarding medication histories, as these data were obtained using questionnaire responses, rather than prescription records.

The present study has several strengths. First, to the best of our knowledge, ours is the first study to evaluate the association between changes in insulin resistance and cognitive function. Thus, our findings may help elucidate the importance of controlling insulin resistance to prevent cognitive decline among older individuals. Second, we analyzed data from the Ansung cohort study, which provides a large study sample, a community-based prospective design, and long-term follow-up data with information regarding potential confounding factors. Third, we adjusted for important covariates, including GDS-K score, education duration, history of diabetes, history of hypertension, and apolipoprotein E ε4 genotype status. Fourth, we excluded patients with high GDS scores to minimize the influence of depressiveness on the MMSE results, as depression can be associated with low MMSE scores^18^. Fifth, we excluded participants with conditions that could influence cognitive function, such as stroke, dementia, depression, and head trauma.

The study also has potential limitations. First, although the current study used data from a large-scale prospective community-based cohort study, only 422 subjects were assessed for cognitive function, which may have limited the assessment of metabolic factors’ effect on cognitive change. Second, the follow-up period may not be sufficient to detect a decrease in cognitive function among individuals with normal baseline cognitive function, and the 6-year change in the MMSE value was relatively small. Third, cognitive function was only evaluated using MMSE, although insulin resistance and hyperglycemia are reportedly more related to verbal performance or executive function. Fourth, increased insulin resistance was associated with a decrease in MMSE during the study, although we did not evaluate whether improvements in insulin resistance prevented cognitive decline. In addition, although the MMSE is a widely used tool for cognitive dysfunction screening, it cannot identify small changes in cognitive function (e.g., cognitive aging) because of a ceiling effect. Therefore, more accurate evaluation of cognitive function must be performed using detailed neurocognitive assessments without ceiling effects.

In conclusion, increased insulin resistance was associated with decreased cognitive function during a 6-year follow-up of older individuals with normal baseline cognitive function. However, baseline HbA1c, baseline BMI, ∆HbA1c, and ∆BMI values were not associated with changes in cognitive function. These relationships were independent of apolipoprotein E ε4 genotype status. Based on our results, further interventional studies are needed to evaluate the effect of controlling insulin resistance on cognitive dysfunction among older individuals.
